# Estimated HIV Incidence in California, 2006–2009

**DOI:** 10.1371/journal.pone.0055002

**Published:** 2013-02-06

**Authors:** Susan Scheer, Shoshanna Nakelsky, Trista Bingham, Mark Damesyn, Dan Sun, Chi-Sheng Chin, Anthony Buckman, Karen E. Mark

**Affiliations:** 1 San Francisco Department of Public Health, HIV Epidemiology Section, San Francisco, California, United States of America; 2 Division of HIV and STD Programs, Los Angeles County Department of Public Health, Los Angeles, California, United States of America; 3 Office of AIDS, California Department of Public Health, Sacramento, California, United States of America; Fundacion Huesped, Argentina

## Abstract

**Introduction:**

Accurate estimates of HIV incidence are crucial for prioritizing, targeting, and evaluating HIV prevention efforts. Using the methodology the CDC used to estimate national HIV incidence, we estimated HIV incidence in Los Angeles County (LAC), San Francisco (SF), and California’s remaining counties.

**Methods:**

We estimated new HIV infections in 2006–2009 among adults and adolescents in LAC, SF and the remaining California counties using the Serologic Testing Algorithm for Recent Seroconversion (STARHS). STARHS methodology uses the BED HIV-1 capture enzyme immunoassay to determine recent HIV infections by testing remnant serum from persons newly diagnosed with HIV. A population-based incidence estimate is calculated using HIV testing data from newly diagnosed cases and imputing for persons unaware of their HIV infection.

**Results:**

For years 2007–2009, respectively, we estimated new infections in LAC to be 2426 (95% CI 1871–2982), 1669 (CI 1309–2029) and 1898 (CI 1452–2344) (p<0.01); in SF for 2006–2009, 492 (CI 327–657), 490 (CI 335–646), 458 (CI 342–574) and 367 (CI 261–473) (p = 0.14); and in the remaining California counties in 2008–2009, 2526 (CI 1688–3364) and 2993 (CI 2141–3846) respectively. HIV infection rates among men who have sex with men (MSM) in LAC were 100 times higher than other risk populations; the SF MSM rate was 3 to 18 times higher than other demographic groups. In LAC, incidence rates among African-Americans were twice those of whites and Latinos; persons 40 years or older had lower rates of infection than younger persons.

**Discussion:**

We report the first HIV incidence estimates for California, highlighting geographic disparities in HIV incidence and confirming national findings that MSM and African-Americans are disproportionately impacted by HIV. HIV incidence estimates can and should be used to target prevention efforts towards populations at highest risk of acquiring new HIV infections, focusing on geographic, racial and risk group disparities.

## Introduction

Approximately 30 years after the initial case of AIDS was reported in the United States [1], the first National HIV/AIDS Strategy (NHAS) was released by the White House in July 2010 [2]. One of the key goals of the NHAS is to reduce the number of new HIV infections by 25 percent by 2015. Information from the national HIV/AIDS surveillance system, including HIV incidence surveillance activities, is needed to evaluate progress towards reducing HIV transmission and to address other goals of the NHAS. In particular, accurate estimates of newly acquired HIV infections are crucial for prioritizing, targeting, and evaluating HIV prevention and disease control efforts. The Centers for Disease Control and Prevention (CDC) implemented a sentinel-based, national HIV Incidence Surveillance (HIS) system in 2004 to monitor the number and rates of new HIV infections in the U.S. and has recently released annual incidence estimates for 2006–2009 [3]. The Los Angeles County Department of Public Health, the San Francisco Department of Public Health, and the California Department of Public Health have served as three of the 25 national HIS sentinel sites since 2004. However, data from the three California HIS sites have not yet been included in the nationwide estimates due to the recency of California’s implementation of name-based HIV reporting in April 2006 compared with other states’ more mature systems.

California, the state that reported the first AIDS cases in the United States [1], continues to be severely impacted both in terms of absolute numbers of infected persons and rates of HIV infection relative to the country as a whole. As of December 31, 2008, there were 103,073 adults and adolescents living with HIV or AIDS in California, second only to New York [4]. Within California, the HIV epidemic has most heavily impacted Los Angeles and San Francisco Counties, representing 37% (36,705 cases) and 14% (14,440 cases), respectively, of the total HIV/AIDS cases living in California as of 2008 [5], [6], [7].

While HIV incidence estimates for the U.S. are crucial for assessing progress towards achieving the goals set forth in the NHAS, states and local jurisdictions must also prioritize and tailor prevention and control efforts to meet the specific needs of their affected populations. By applying the statistical methodology CDC uses to estimate HIV incidence for the nation [3], [8], we have estimated HIV incidence among persons aged 13 years and older for Los Angeles, San Francisco, and California’s remaining counties so that these estimates can be used to evaluate the success of and ongoing need for HIV prevention services. In addition, these estimates can serve as a baseline from which to measure local progress achieving NHAS goals.

## Methods

The Los Angeles County and San Francisco Departments of Public Health conduct HIS activities for their respective counties and the California Department of Public Health oversees HIS activities for the remaining counties in the state. Each jurisdiction estimated the number of persons aged 13 years or older newly infected with HIV in their geographic areas using Serologic Testing Algorithm for Recent Seroconversion (STARHS) and the stratified extrapolation method previously reported and described [3], [8]. Briefly, the STARHS methodology uses the BED HIV-1 capture enzyme immunoassay (BED) [9] to determine the number of recent HIV infections by testing remnant serum specimens from persons newly diagnosed and reported to HIV/AIDS surveillance systems. The stratified extrapolation method allows for the calculation of a population-based HIS estimate by using available HIV testing history data from newly diagnosed cases aged 13 years for older (age range 13–88 years) and imputing this information for persons who remain unaware of their HIV infection. Following the first application of the stratified extrapolation method in 2008 [10], two major components have been modified: 1) the method for estimating the probability of being detected in the STARHS recency period and 2) the method for addressing information on HIV mode of transmission. The details of these modifications have been previously published [3]. We used the revised methodology to calculate HIV incidence estimates for years 2006–2009 in each of the three California HIS jurisdictions.

HIV incidence estimates are based on BED test results and HIV testing and treatment history from adolescent and adult cases reported to the Enhanced HIV/AIDS Reporting System (eHARS) across all three jurisdictions between 2006 and 2009. For each newly diagnosed HIV case reported in eHARS, we sought to obtain a remnant serum specimen for BED testing from the confirmatory HIV Western blot test. We collaborated with public health, hospital-based and commercial laboratories to retrieve and ship sera to the national CDC-designated STARHS laboratory. In accordance with CDC recommendations, HIV incidence estimates were not calculated for years in which a STARHS result was not available for at least 15% of the newly diagnosed HIV cases.

Individual variation in the development of antibodies and other factors can affect the validity of BED results. To adjust for this variation, HIS attempts to incorporate information about the HIV testing and antiretroviral treatment history of newly diagnosed cases to produce more accurate estimates. To improve the statistical estimation of new HIV infections, each jurisdiction collects date of first positive HIV test, date of last negative HIV test, the number of negative HIV tests within 24 months of the first positive, and any use of antiretroviral therapy within the 6 months prior to the positive test.

Statistical programs developed by CDC using SAS 9.2 [11] software were tailored for local use to calculate HIV incidence estimates. These SAS programs use multiple imputation techniques to fill in plausible values for data missing on those who do not seek HIV testing and then to impute missing BED test results and HIV testing history information. The incidence calculation is based on the probability of obtaining an HIV test within one year of infection, the probability of being BED tested, and the probability of the BED test being classified as a recent infection. When missing values have been imputed using demographic information from eHARS, a population-based HIV incidence estimate is calculated. If a sample size of new HIV cases was adequate, HIV incidence estimates for specific sub-populations were calculated. Pairwise comparisons were tested using z-tests. Linear tests for trend in HIV incidence by year were conducted via tested using random effects meta regression using the z-statistic [12]. Incidence rates were calculated separately for each of the three jurisdictions using denominators obtained from population estimates of the 2010 California Department of Finance [13] for age, sex, and race/ethnicity. The denominators include all individuals aged 13 years and older residing in Los Angeles County, San Francisco County and all other California counties. Local estimates of the size of the population of men who have sex with men (MSM) were used to calculate HIV incidence rates for this population [6], [14].

This analysis was considered exempt from Institutional Review Board review in accordance with the Code of Federal Regulations Regarding Protection of Human Subjects, 45 CFR 46.101 (b) as the analyses were conducted using existing data and specimens collected through routine public health surveillance activities and the information is recorded by the investigator in such a manner that subjects cannot be identified directly or through identifiers linked to the subjects.

## Results

Table 1 provides details on the number of HIV/AIDS cases diagnosed and reported and the completeness of HIV testing history and BED test results, data elements used to estimate HIV incidence by year in each of the three California HIS jurisdictions. As the HIS systems matured in each jurisdiction, the proportion with complete testing and treatment history and BED results improved. For all years San Francisco County reported the highest completeness rate for HIV testing history collection and BED test results followed by Los Angeles and all other California counties. Following CDC’s recommendation to restrict the calculation of incidence to years in which BED results were available for at least 15% of newly diagnosed HIV cases, we calculated incidence in San Francisco County for 2006–2009, in Los Angeles County for 2007–2009 and the remaining California counties for 2008–2009.

**Table 1:** Number of reported newly diagnosed HIV/AIDS cases and non-AIDS HIV diagnoses, with completeness data for HIV testing history and BED test results, for the three HIV Incidence Surveillance areas in California, 2006–2009.

	Los Angeles County				San Francisco City and County				All California counties excluding Los Angeles and San Francisco Counties			
Year of Diagnosis	Newly Diagnosed HIV/AIDS cases	Non–AIDS HIV Diagnoses	Testing History^1^	BED Result^2^	Newly Diagnosed HIV/AIDS cases	Non–AIDS HIV Diagnoses	Testing History^1^	BED Result^2^	Newly Diagnosed HIV/AIDS cases	Non–AIDS HIV Diagnoses	Testing History^1^	BED Result^2^
	N	n	n (%)	n (%)	n	n	n (%)	n (%)	n	n	n (%)	n (%)
2006	2647	1941	477 (18%)	244 (13%)	557	405	338 (61%)	165 (41%)	2962	1839	365 (12%)	180 (10%)
2007	2662	2030	819 (31%)	474 (23%)	565	413	386 (68%)	213 (52%)	2994	1942	418 (14%)	234 (12%)
2008	2388	1706	1044 (44%)	715 (42%)	526	393	394 (75%)	257 (65%)	2853	1874	525 (18%)	466 (25%)
2009	2138	1509	1208 (57%)	555 (37%)	483	357	394 (82%)	248 (69%)	2753	1740	826 (30%)	447 (26%)

1 Defined as having an answer to the question “Have you ever tested negative for HIV in the past”.

2 BED results among Non-AIDS HIV diagnoses.

We estimate that there were approximately 2426 new HIV infections (95% CI 1871–2982) among adults and adolescents in Los Angeles County in 2007, 1669 (95% CI 1309–2029) and 1898 (95% CI: 1452–2344) new infections in 2008 and 2009, respectively. The decline in incidence between 2007 and 2009 was statistically significant (trend p<0.01). The rate of new HIV infection in Los Angeles County (per 100,000 residents) was 29 in 2007, 20 in 2008, and 23 in 2009 (Table 2).

**Table 2:** Estimated number and rate per 100,000 of new HIV infections by HIV Incidence Surveillance area and year.

	Los Angeles County		San Francisco City and County		All California counties excluding Los Angeles and San Francisco Counties		
Year of Diagnosis	Estimated Incident HIV Cases (95% CI)^1^	Rate (95% CI)	Estimated Incident HIV Cases (95% CI)^1^	Rate (95% CI)	Estimated Incident HIV Cases (95% CI)^1^	Rate (95% CI)	
2006	Not presented^2^		492	70	Not presented^2^		
			(327–657)	(47–94)			
2007	2426	29	490	70	Not presented^2^		
	(1871–2982)	(22–36)	(335–646)	(48–92)			
2008	1669	20	458	65	2526		13
	(1309–2029)	(16–24)	(342–574)	(49–82)	(1688–3364)		(9–17)
2009	1898	23	367	52	2993		15
	(1452–2344)	(17–28)	(261–473)	(37–67)	(2141–3846)		(11–20)

1 CI, Confidence Interval.

2 Incidence estimate not calculated due to incomplete data.

In 2006, there were an estimated 492 new HIV infections among adults and adolescents (95% CI 327–657) in San Francisco, and 490 new infections (95% CI: 335–646), 458 new infections (95% CI: 342–574), and 367 new infections (95% CI 261–473) in 2007, 2008, and 2009, respectively. The change in incidence between 2006 and 2009 was not statistically significant (trend p = 0.14). The rate of new HIV infection in San Francisco (per 100,000 residents) was 70 in 2006, 70 in 2007, 65 in 2008 and 52 in 2009 (Table 2).

There were 2526 new HIV infections among adults and adolescents in the remaining California counties in 2008 (95% CI: 1688–3364) and 2993 new infections (95% CI 2141–3846) in 2009. The rate of new HIV infection (per 100,000 residents) was 13 in 2008 and 15 in 2009 (Table 2).

The estimated HIV incidence rates (per 100,000 residents) among sub-populations in Los Angeles and San Francisco counties are presented in Tables 3 and 4. In both counties and in every year when stratification was possible, MSM had the highest rates of HIV infection: 447 and 493 in Los Angeles in 2008 and 2009, respectively, and 807, 783, 788, and 626 in San Francisco in 2006, 2007, 2008 and 2009, respectively. The rates among MSM in Los Angeles were 9 to 111 times the rate of HIV infection in other demographic groups in Los Angeles and the MSM rate in San Francisco was 3 to 18 times higher than rates in all other demographic groups in San Francisco. African Americans appeared to experience higher rates of HIV infection compared with whites and these findings reached statistical significance in Los Angeles. In Los Angeles, the rate of HIV infection among African Americans in 2008 and 2009 (44 and 56 per 100,000, respectively) was more than twice that of both whites (17 and 20 per 100,000, respectively) and Latinos (20 and 23 per 100,000, respectively). In San Francisco, the higher rate of HIV infection among Latinos compared to whites in 2008 and African Americans compared to whites in 2009 was not statistically significant. In both Los Angeles and San Francisco, persons 40 years or older had lower rates of HIV infection compared to younger age groups, although in San Francisco this difference was not statistically significant.

**Table 3:** Estimated rate of new HIV infections by sex, race/ethnicity, age group and mode of transmission, Los Angeles County, 2008–2009.

	2008		2009	
	Rate (per 100,000)	(95% CI)	Rate (per 100,000)	(95% CI)
**Sex at Birth**				
Male	36	(27–44)	38	(28–47)
Female	4	(2–7)	8	(2–13)
**Race/ethnicity**				
White	17	(12–23)	20	(12–27)
Black	44	(29–59)	56	(34–78)
Latino	20	(14–27)	23	(15–31)
Other	**	**	**	**
**Age at Infection (years)**				
13–29	25	(18–31)	27	(20–35)
30–39	34	(23–46)	40	(24–55)
40+	12	(8–17)	14	(9–19)
**Mode of Transmission**				
MSM^1^	447	(339–555)	493	(369–617)
HET/IDU/OTH^2^	4	(1–6)	5	(1–8)

1 MSM includes MSM-IDU.

2 Heterosexual/Injection Drug Use/Other mode of transmission.

** Incidence estimate not calculated due to incomplete data.

**Table 4:** Estimated rate of new HIV infections by sex, race/ethnicity, age group and mode of transmission, San Francisco County, 2006–2009.

	2006		2007		2008		2009	
	Rate (per 100,000)	(95% CI)	Rate (per 100,000)	(95% CI)	Rate (per 100,000)	(95% CI)	Rate (per 100,000)	(95% CI)
**Sex at Birth**								
Male	128	(84–173)	125	(84–166)	123	(90–155)	98	(69–128)
Female	**	**	**	**	**	**	**	**
**Race/ethnicity**								
White	86	(55–118)	85	(5–119)	75	(50–99)	55	(36–74)
Black	**	**	**	**	117	(38–199)	181	(0–375)
Latino	68	(28–109)	102	(28–178)	148	(61–233)	68	(26–110)
Other	**	**	**	**	**	**	**	**
**Age at Infection (years)**								
13–29	79	(45–114)	118	(57–179)	97	(55–138)	80	(45–115)
30–39	84	(48–120)	85	(51–120)	88	(55–121)	70	(34–107)
40+	59	(26–93)	44	(18–70)	42	(25–59)	34	(19–49)
**Mode of Transmission**								
MSM^1^	807	(536–1078)	783	(517–1047)	788	(579–998)	626	(450–800)
HET/IDU/OTH^2^	**	**	**	**	**	**	**	**

1 MSM includes MSM-IDU.

2 Heterosexual/Injection Drug Use/Other mode of transmission.

** Incidence estimate not calculated due to incomplete data.

## Discussion

We report the first HIV incidence estimates for California using the revised stratified extrapolation approach for incidence estimation developed by the CDC. These estimates highlight the geographic disparity in new HIV infections throughout California. San Francisco, for example, had the fewest number of new infections in all reported years between 2006 and 2009, but had the highest rate per 100,000 than any other California counties including Los Angeles County. The rate of new HIV infections in San Francisco was two to three times higher than the rate of new HIV infections in Los Angeles County and four to five times higher than in the remaining California counties. Rates of new HIV infections in Los Angeles were approximately 1.5 times higher than rates in the remaining California counties. Overall, Los Angeles saw a statistically significant decline in HIV incidence while San Francisco did not.

Compared to national HIV incidence estimates for the years 2006–2009, the rate of new infections in San Francisco was on average three times higher than national rates that ranged from a low of 19.0 (95% CI:16.6–21.3) to a high of 22.5 (95% CI: 19.7–25.3) new infections per 100,000 in these years [3]. The HIV incidence rate in Los Angeles during 2007–2009 was nearly identical to the national rates while the incidence rate in the remaining California counties was somewhat lower than the national rate. Also similar to national estimates, San Francisco and Los Angeles counties reported their highest incidence rate in 2007. Analyses of HIV incidence among specific population groups in San Francisco and Los Angeles counties confirms national HIV incidence findings demonstrating that MSM and African Americans have been disproportionately impacted by HIV disease. These comparisons were not possible in the remaining California counties due to small subpopulation sample sizes and incomplete BED test results.

Similar to national HIV incidence estimates, the refinements in the statistical methodology resulted in a lower estimate of HIV incidence than previously published. For San Francisco, these new estimates of HIV incidence are 50%, 36% and 25% lower than previously published estimates for 2006, 2007, and 2008, respectively [15]. A significant proportion of the difference found between the previous and new estimates may be attributed to refinement in the stratified extrapolation methodology used for calculating incidence. For example, the changes to the STARHS recency period would account for approximately a 4% decrease in the estimate and changes to the methodology to address the concern that the likelihood of testing for HIV within one year was previously overestimated could account for an additional 7% decrease in the estimate [3].

There are a number of possible limitations that should be noted in our estimation of HIV incidence, most of which are implicit in the assumptions of the statistical model and the data elements available from the HIV/AIDS surveillance system including BED test results and HIV testing history. These limitations are discussed in detail in the CDC’s presentation of national HIV incidence estimates [3]. Completeness of both BED test results and HIV testing history in the three California jurisdictions, while showing improvement over time, varied by jurisdiction and continued to be inadequate to calculate incidence calculations in some years and subpopulations. We were unable to calculate HIV incidence in years or in subpopulations in which the completeness of BED testing was less than 15%. San Francisco, for example, experienced a substantially higher BED specimen ascertainment than the other sites due in large part to the relatively high proportion (approximately 50%) of newly diagnosed HIV cases testing at the two laboratories run by the San Francisco Department of Public Health, which made specimens for BED testing more readily available State-wide legislation providing health departments with specific authority to obtain remnant blood samples from newly diagnosed HIV cases, rather than relying on the voluntary participation from private laboratories, would likely increase the completeness of BED specimen ascertainment and testing. In the absence of a state-wide law, it is necessary for each HIS jurisdiction to collaborate with local laboratories to develop procedures that alleviate the potential burden of specimens submission and ultimately ensure more complete specimen storage and shipping of remnant sera.

The HIV incidence estimates presented here highlight the continued need for enhanced prevention programs, HIV testing, and linkage-to-care interventions in California to reach the National HIV/AIDS Strategy’s goals of reducing HIV incidence by 25% by 2015. Increasing evidence of HAART as an effective means of preventing HIV transmission through viral suppression has been presented at the community level [16], among serodiscordant heterosexual couples [17], [18], in mother-to-child transmission [19] and in mathematical models [20], [21]. Therefore, allocation of resources to increase availability of and retention in HIV care is an important step to slow the spread of the epidemic in California. HIV incidence estimates along with HIV prevalence estimates can and should be used to target HIV prevention and care efforts towards the populations at highest risk of acquiring new HIV infections with a focus on addressing the significant geographic, racial and risk group disparities identified.
